# Development of a modular neonatal neurological examination: from a short screening test to a detailed assessment of specific aspects of neurological function

**DOI:** 10.1007/s00431-026-07049-4

**Published:** 2026-05-18

**Authors:** Eugenio Mercuri, Daniela Ricci, Frances M. Cowan, Leena M. Haataja, Domenico M. Romeo

**Affiliations:** 1 Pediatric Neurology Unit, Fondazione Policlinico Universitario A. Gemelli IRCCS, Largo Gemelli, 00168 Rome, Italy; 2 Pediatric Neurology Unit, Università Cattolica del Sacro Cuore Roma, Rome, Italy; 3 National Centre of Services and Research for the Prevention of Blindness and Rehabilitation of Low Vision Patients - IAPB Italia Onlus, Rome, Italy; 4 Department of Paediatrics, Imperial College, London, UK; 5 Pediatric Neurology, Children’s Hospital, University of Helsinki and Helsinki University Hospital, Helsinki, Finland

**Keywords:** Neurological examination, Neonatal neurological examinations, Modular approach, Additional modules

## Abstract

The neurological examination of the newborn is often perceived as difficult. This paper describes the development of a modular approach, based on the Hammersmith Neonatal Neurological Examination, including a short proforma for routine use, the standard examination and two add-on modules. We report details of the single modules with description of their use in different scenarios.

*Conclusions*: The modular approach provides a number of options for performing an appropriate level of clinical neurological examinations in all newborns accommodating different needs in clinical and research settings.

**Table:** 

**What is Known:** • *Neonatal neurological examinations are often not performed as perceived as difficult.* • *D*etailed examinations are a burden in routine clinical practice and not always needed.	
**What is New:** • *A modular approach, including a short examination, can be used in clinical routine.* • *The additional modules, when needed, can help in differential diagnosis.*

**What is Known:**

• *Neonatal neurological examinations are often not performed as perceived as difficult.*

• *D*etailed examinations are a burden in routine clinical practice and not always needed.

**What is New:**

• *A modular approach, including a short examination, can be used in clinical routine.*

• *The additional modules, when needed, can help in differential diagnosis.*

In an ideal world each newborn should receive a neurological examination but this is often not the case in clinical practice [1]. A neurological examination is particularly relevant in preterm infants, who are at higher risk of developing brain lesions, and in all infants presenting with signs suggestive of neurological involvement [2]. It should also be routinely performed as part of the examination before discharge to allow early detection of abnormal findings and to direct further investigations. In the real world, a structured examination is often perceived as difficult and it is often poorly performed and recorded.

The Hammersmith Neonatal Neurological Examination (HNNE), originally developed by Dubowitz and Dubowitz in the early 80 s [3] and subsequently revised [4], is easily performed, can be performed even in incubators and has been proven to be able to detect neurological abnormalities in both preterm and full-term infants. Over the years the HNNE has been adapted to accommodate emerging needs, including a shorter examination to be used as a routine tool that has now been validated for both preterm and full-term infants [4, 5]. There has also been an increasing need for additional tools to provide more details on specific aspects of neurological findings found to be abnormal on the standard HNNE, that has prompted the development of additional modules for floppy infants [6] or those with abnormal visual function [7]. While the increased number of available forms might appear to be an extra burden for the clinician, in practice they are part of a modular approach that can accommodate different needs.

In this paper we describe our modular approach to using the HNNE in several settings, from routine practice to a more accurate assessments in clinical and research settings.

## Step 1: short module for routine examination

This module was developed to be used as part of the routine examination at discharge in all newborns [4]. Although the HNNE can be easily performed in a short time, it is often considered too lengthy in the context of the limited time available for examining all newborns in the maternity ward. This shorter module addresses this issue by selecting a reduced number of items that can fit ona A4 page. The screening assessment tool includes only 11 of the 34 items included in the HNNE and five abnormal signs. The selection was based on the need to maintain a fair representation of all the sections in the HNNE, identifying the items that in our experience were more sensitive to detecting possible “warning signs” of neurological abnormalities.

The scoring system is also simplified. The 11 items are set out in 3 columns based on the frequency distribution of findings obtained in low-risk term infants in the original studies on the HNNE, identifying, for each item, the findings falling outside the 90% centile that could be potentially considered as “warning signs.” In this format the central column includes the spectrum of neurologic findings falling within the normative reference range (90%) that could therefore be considered as optimal while those falling outside the 90% centile are in two lateral columns headed “warning signs.”

This module, originally developed as a screening tool for full-term infants, was subsequently validated for preterm infants at term age [5]. Analyzing the frequency distribution of findings obtained in low-risk preterm infants, it was found that for all 11 items the findings falling outside the 90th percentile in the low-risk preterm cohort were the same as those previously found for full-term infants.

The form also includes 5 abnormal signs that are recorded using a choice of yes/no.

As a rule of thumb, infants with more than one warning sign should be examined with a full neurological examination using the HNNE. The short examination and its recording generally do not take longer than 5 min.

## Step 2: full neurological examination (HNNE)

This module should be used in all newborns who have more than one “warning sign” on the short assessment described above or in those with known neurological risk or with overt neurological signs. The HNNE in its current version has been used for over 25 years [4, 8] and includes various aspects of neurologic function, such as posture, tone, abnormal tone patterns, reflexes, motility, and behavior.

The examination can be used at any gestational or postnatal age up to 2 months post-term age thus enabling the recording of maturational changes occurring with increased gestational/postnatal age as well as abnormal findings observed in newborns with neurological impairment. The examination is presented on a recording sheet (proforma) on which there are simple figure diagrams for each item making the recording (and ultimately the scoring) easy. The full examination and its recording generally do not take longer than 10–15 min.

An optimality score was developed to provide a more accurate quantification to be mainly used in research settings [8]. The optimality score was initially only based on reference data from full-term newborns but the range of findings in preterm of different ages has subsequently also become available [9–13].

The examination has been used worldwide and provides a general overview of the neurological status of the newborn infant both in preterm and full-term newborns, also enabling the identification of specific aspects of neurological function, such as generalized hypotonia or possible cerebral visual impairment, that should be further explored.

## Step 3: add-on modules

**Fig. 1:**
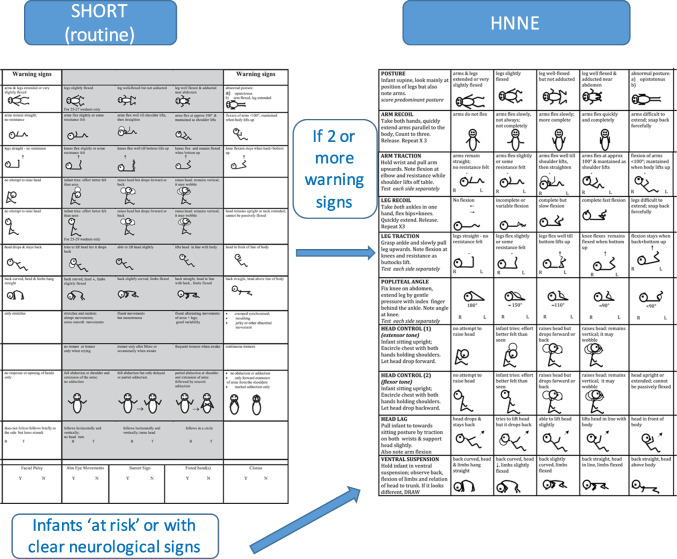

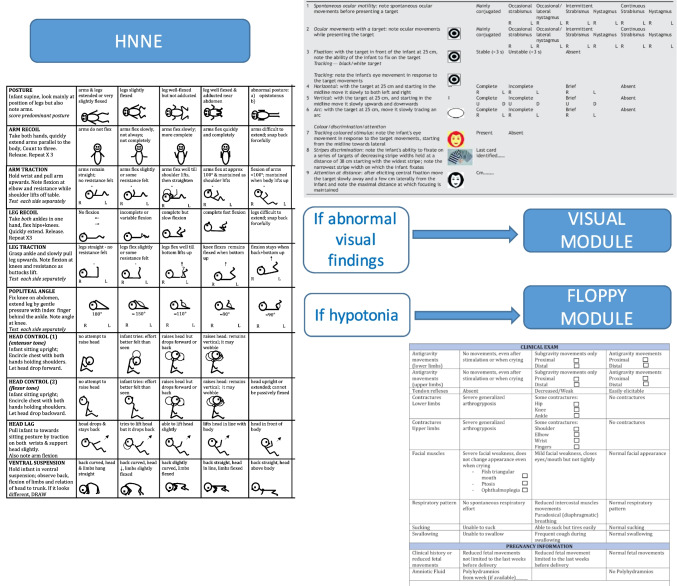
Flowchart of the use of the different proforma

To avoid increasing the time needed to complete the HNNE, additional items were incorporated in add-on modules that are not for routine used together but are available when the abnormal findings on the HNNE suggest the need for a more detailed assessment of these aspects (Fig. 1).

### Add-on module for neonatal visual assessment

The HNNE includes two items assessing ocular movements and the ability to fix and follow and one on visual alertness. The module for neonatal visual assessment was designed to provide more detailed information on visual function [7]. Although visual function in the newborn infants is still predominantly subcortical and many aspects are very immature compared to older infants, there are a number of aspects that can still be assessed and allow early identification of abnormalities that may benefit from early intervention [7]. The additional module includes 9 items assessing ocular movement (spontaneous and using a visual target), reaction to a colored target, discrimination of stripes with increasing spatial frequency, and attention at distance. The module, which was validated in low-risk term newborns can also be applied in infants as young as 32 weeks gestation. The assessment and its recording generally do not take longer than 5–10 min.

The additional module for visual function has been used in infants with brain lesions and those at risk of cerebral visual impairment showing a very high sensitivity for the detection of early visual abnormalities.

### Module for floppy neonate

This module was specifically designed to be used in newborns in whom the first section of the HNNE, assessing posture and tone, shows a generalized reduction in tone. Neonatal hypotonia is a common finding with multiple causes, including peripheral or central nervous system involvement, syndromic or metabolic disorder.

This module provides a number of additional items that can help in differentiating central from peripheral neurological involvement or in identifying other signs suggestive of a metabolic or genetic etiology, thus guiding appropriate investigations. These items include assessment of antigravity movements and contractures that are known to be sensitive and specific indicators of neuromuscular disorders [2, 14], but also the assessment of possible dysmorphic features that are more often associated with genetic etiologies. The module also includes items (e.g. a history of reduced fetal movements or polyhydramnios) that may also suggest a neuromuscular etiology. This add-on module can be easily performed and recorded in 5 min and does not require extra equipment.

The module has been validated in low-risk newborns and in a cohort with neonatal hypotonia [6], showing a very high sensitivity for detecting neurologic findings suggestive of different etiologies, thus facilitating differential diagnosis. It has also recently been used to detect signs of disease in newborns with spinal muscular atrophy identified by neonatal screening [15].

## Conclusions

The modular approach reported here provides the possibility to perform an appropriate level of clinical neurological examinations in all newborns, giving a number of options accommodating different needs in clinical and research settings. While the short routine examination facilitates the clinician who finds it difficult to perform the full examination in the busy routine on the ward, the other modules provide additional information that can be relevant for differential diagnosis and early intervention.

## Data Availability

No datasets were generated or analysed during the current study.
